# Longevity of protective immune responses induced by a split influenza A (H7N9) vaccine mixed with MF59 adjuvant in BALB/c mice

**DOI:** 10.18632/oncotarget.20064

**Published:** 2017-08-08

**Authors:** Huilin Ou, Wei Yao, Dongshan Yu, Tianhao Weng, Frederick X.C. Wang, Xiaoxin Wu, Haibo Wu, Linfang Cheng, Xiangyun Lu, Nanping Wu, Honglin Chen, Lanjuan Li, Hangping Yao

**Affiliations:** ^1^ State Key Laboratory for Diagnosis and Treatment of Infectious Diseases, Collaborative Innovation Center for Diagnosis and Treatment of Infectious Diseases, The First Affiliated Hospital, Zhejiang University School of Medicine, Hangzhou, China; ^2^ Zhejiang Tianyuan Bio-Pharmaceutical Co., Ltd., Hangzhou, China; ^3^ Department of Bioengineering, Erik Jonsson School of Engineering and Computer Science, The University of Texas at Dallas, Dallas, Texas, USA; ^4^ State Key Laboratory for Emerging Infectious Diseases, Carol Yu Centre for Infection, The University of Hong Kong, Hong Kong, China

**Keywords:** H7N9, adjuvant vaccine, MF59, immunogenicity, protective immune responses

## Abstract

The influenza virus is a serious threat to public health worldwide. A novel avian influenza A (H7N9) virus with a mortality rate of approximately 30% has been identified as an unusually dangerous virus for humans by the World Health Organization. Pathogenic H7N9 continue to represent a public health concern, and several candidate vaccines are currently in development. We generated candidate H7N9 vaccine strains using reverse genetics, consisting of hemagglutinin and neuraminidase genes derived from a human H7N9 virus and the remaining genes from the PR8 (A/PuertoRico/8/34 (H1N1)) virus. This H7N9 vaccine exhibited superior efficacy when combined with MF59 compared to other adjuvants. Immunized BALB/c mice were followed to determine the duration of the protective immune response. Antibody levels decreased to between one-half and one-eighth of the peak values four months after the final dose of the vaccine. Previously vaccinated mice received an A/Zhejiang/DTID-ZJU01/2013 H7N9 challenge six months post-vaccination, and all remained protected. We also verified that MF59 enhanced the HI, MN, and IgG antibody titers to influenza antigens. The humoral immune response and Th2 cytokine production following influenza challenge was potently induced in the animals that received the split vaccine. Therefore, the split H7N9 influenza vaccine with the MF59 adjuvant could effectively induce antibody production and protect mice from H7N9 virus challenge even after six months.

## INTRODUCTION

Human influenza virus infections with the H7N9 strain were first reported in China in February 2013 [1], and there have been five subsequent waves of infection. Moreover, total of 1486 laboratory-confirmed cases of human infection with avian influenza A (H7N9) viruses in China, including at least 540 deaths, had been reported to the World Health Organization (WHO) as of May 23, 2017 [2]. The virus can cause rapidly progressive pneumonia, often complicated by extrapulmonary disease associated with hypercytokinemia, as well as significant mortality and morbidity [3].

There is minimal pre-existing natural immunity against the new H7N9 avian strain in human populations. Therefore, influenza vaccination, which has been used for more than 60 years, is the primary strategy used for influenza prevention and control. However, several H7 influenza vaccines in clinical development have limited protective efficacy due to the poor immunogenicity of the H7 hemagglutinin (HA) in humans [4–6]. To overcome this challenge, the addition of adjuvants has been used to enhance immune responses.

Currently, vaccine production greatly benefits from molecular biology methods. Particularly useful is the application of reverse genetics, which allows for the removal of pathogenic traits at the plasmid stage to generate novel vaccines and vaccine vectors [7, 8]. The reassortant candidate virus was constructed using reverse genetics technologies that contains the HA and neuraminidase (NA) glycoproteins of A/Zhejiang/DTID-ZJU01/2013 (H7N9), with the remaining 6 genes derived from PR8. It has the advantage of rapid preparation and rapid amplification. Accordingly, we verified that the recombinant clones harbor the desirable characteristics, including lower virulence and transmissibility, as a vaccine strain [9]. Additionally, the oil-in-water adjuvant MF59, which we have paired with our split virus vaccine, has been reported to be safe, well-tolerated, and able to improve the antibody response, permit dose sparing, and lower the antigen dose required to induce protection [10, 11]. Besides, it has been reported the addition of MF59 to split or subunit influenza vaccines can induce much more improvement than the classic immunoadjuvant alum [12].

The vast majority of vaccines are preclinically evaluated for only short-term efficacy over duration of a few weeks. While these vaccines were found to be efficacious shortly after vaccination, it is also vital to assess the duration of the induced protective antibodies following vaccination. Additionally, the immune induction mechanisms of the research vaccine should also be evaluated. In the present study, BALB/c mice were housed in strict isolation for up to six months, and we report the extension of our earlier findings, including the level of immunity following viral challenge in these mice six months after vaccination.

## RESULTS

### Evaluation of antibody responses

**Figure 1:**
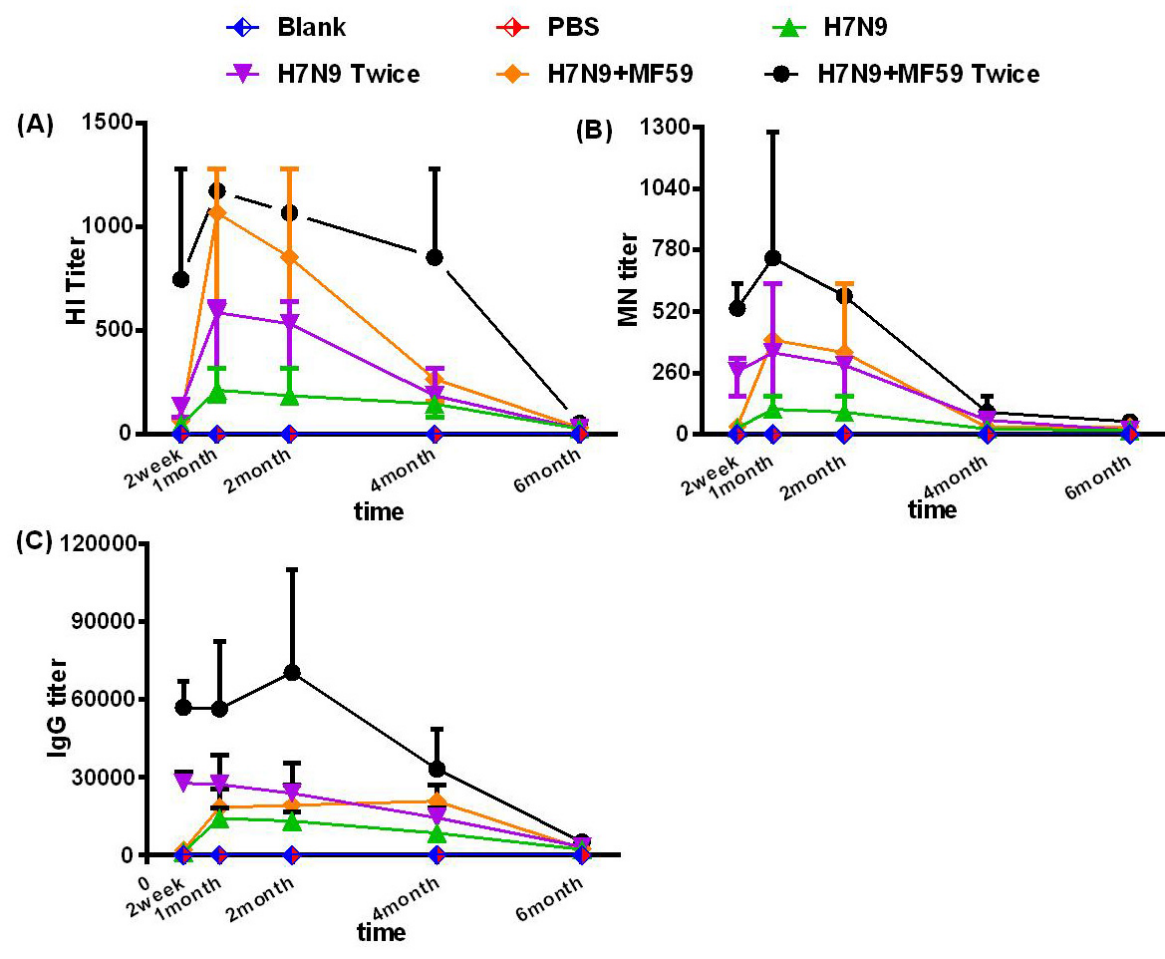
Measurements of the antibody responses **A.** HI antibodies, **B.** MN antibodies, and **C.** IgG responses of all six groups measured at two weeks, and one, two, four, and six months after the last immunization. Each dot represents the geometric mean titer. Each mouse was intramuscularly injected with various immune formulations: Groups 1 and 2 were treated with nothing and PBS, respectively; Groups 3 and 4 were immunized with one dose 3 μg HA and one dose 3ug HA plus 0.05 mL MF59, respectively; Groups 4 and 6 were immunized with two doses of 3 μg HA and two doses 3 μg HA plus 0.05 mL MF59, respectively with a two-week interval.

Antibody responses in the sera samples of all six groups over the six-month study period are presented in Figure 1. Two weeks after the first vaccination, the geometric mean titers (GMTs) of the hemagglutinin inhibition (HI) titers in the groups immunized with HA antigen (Groups 3 and 5) were all within the range of 10-80, while all microneutralization (MN) titers were below 1:40. The HI, MN, and IgG titers reached a level of significance one month after the first immunization, which was 4 to 16 times higher than the titer of the two week serum sample; this peak persisted at least into the second month. In Groups 4 and 6, the antibody titers reached a relatively high level two weeks after the second immunization, also 1 month based on the first immunization time. For all the groups vaccinated with the HA antigen (Groups 3 − 6), antibody titers in the four month serum samples had regressed rapidly (two months *vs*. four months: HI titer, *p* = 0.003; MN titer, *p* < 0.001; IgG titer, *p* = 0.048). Antibody titers in the 6-month serum samples were virtually undetectable.

The mean peak antibody titer was substantially higher in the groups that received two-doses of the vaccine (Group 4: HI titer 1:570; MN titer 1:320; IgG titer 1:27549; Group 6: HI titer 1:1140; MN titer 1:718; IgG titer 1:60880), compared with that of the groups that received only one dose (Group 3: HI titer 1:202; MN titer 1:101; IgG titer 1:13800; Group 5: HI titer 1:1016; MN titer 1:359; IgG titer 1:17644). In comparing the antibody titers detected at the same time, we observed that the second boost significantly increased the immune response (Group 3 *vs*. Group 5 HI titer, *p* < 0.001; MN titer, *p* = 0.003; IgG titer, *p* = 0.172; Group 4 *vs*. Group 6 HI titer, *p* = 0.004; MN titer, *p* < 0.001; IgG titer, *p* = 0.008). From the above results, we also noted that the peak mean antibody titers were dramatically improved by the addition of the MF59 adjuvant. It is a pity that neither the second dose nor the addition of the MF59 adjuvant failed to boost the antibody responses into the fourth month or prevent the depletion of the responses at sixth months.

### Cellular immune responses in each experimental group

**Figure 2:**
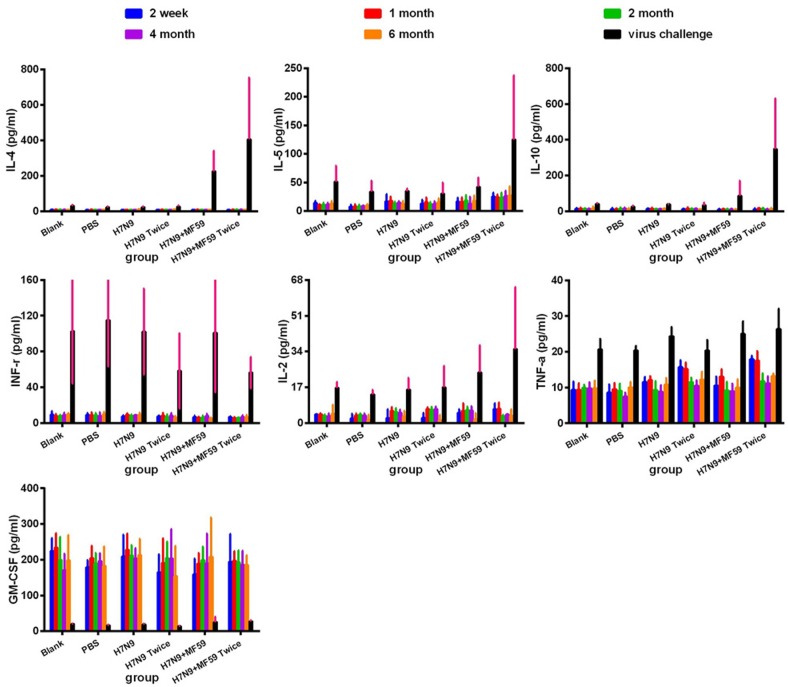
Systemic expression of Th1 (IFN-γ and IL-2), Th2 (IL-4, IL-5, and IL-10) and other cytokines (TNF-α) in experimental groups Group 1 (Blank), Group 2 (PBS), Group 3 (one dose 3 μg HA), Group 4 (one dose 3 μg HA plus 0.05 mL MF59), Group 5 (two doses 3 μg HA), and Group 6 (2 doses 3 μg HA plus 0.05 mL MF59), as measured using a multiplex Luminex LiquiChip. The cytokine expression profiles were measured in the serum of BALB/c mice at two weeks, one, two, four, and six months after the last immunization, and one week after viral challenge.

Figure 2 shows the systemic levels of cytokines at different detection time points in the serum. The cytokines IL-4, IL-5, and IL-10 were produced primarily by Th2 cells. There were also low, but detectable levels of IL-5 and IL-10 in the serum. Vaccination triggered the greatest concentration of IL-4 in the two-dose H7N9+MF59 group (*p* < 0.001); however, there was no statistical difference between the other five groups (*p* = 0.19). Similarly, there was no statistical difference in the levels of IL-5 among all these groups (*p* = 0.277). While IL-4 was below the detection limit (1.57 pg/mL) the level of all three cytokines increased following viral challenge. Levels of IL-4, IL-5, and IL-10 were the greatest in the two-dose H7N9+MF59 group (Group 6) among all experimental groups following viral challenge (IL-4, *p* = 0.003; IL-5, *p* = 0.006; and IL-10 *p* < 0.001). The addition of the MF59 adjuvant to the vaccine resulted in a substantial increase in Th2 cytokine production after virus inoculation (IL-4, *p* = 0.001; IL-5 *p* = 0.044; and IL-10 *p* = 0.014). In contrast, the levels were not statically different between the one-dose groups (Groups 3 and 5) and the two-dose groups (Groups 4 and 6) following viral challenge (IL-4, *p* = 0.511; IL-5, *p* = 0.311; IL-10, *p* = 0.222).

The cytokines IFN-γ and IL-2 are primarily produced by Th1 cells. Low level secretion of the above two cytokines was maintained throughout the period following immunization. The addition of the MF59 adjuvant to the vaccine (Groups 5 and 6) resulted in remarkable changes to the level of IL-2 following viral challenge (*p* = 0.044), but also resulted in lower levels of IFN-γ production following viral challenge (*p* = 0.524). GM-CSF is mainly secreted by activated macrophages, activated T cells, and endothelial cells [13, 14]. Surprisingly, the viral challenge resulted in a sharp decline in the GM-CSF response, which remained at high levels before the viral challenge. There was no significant difference among the six groups (*p* = 0.546).

**Figure 3:**
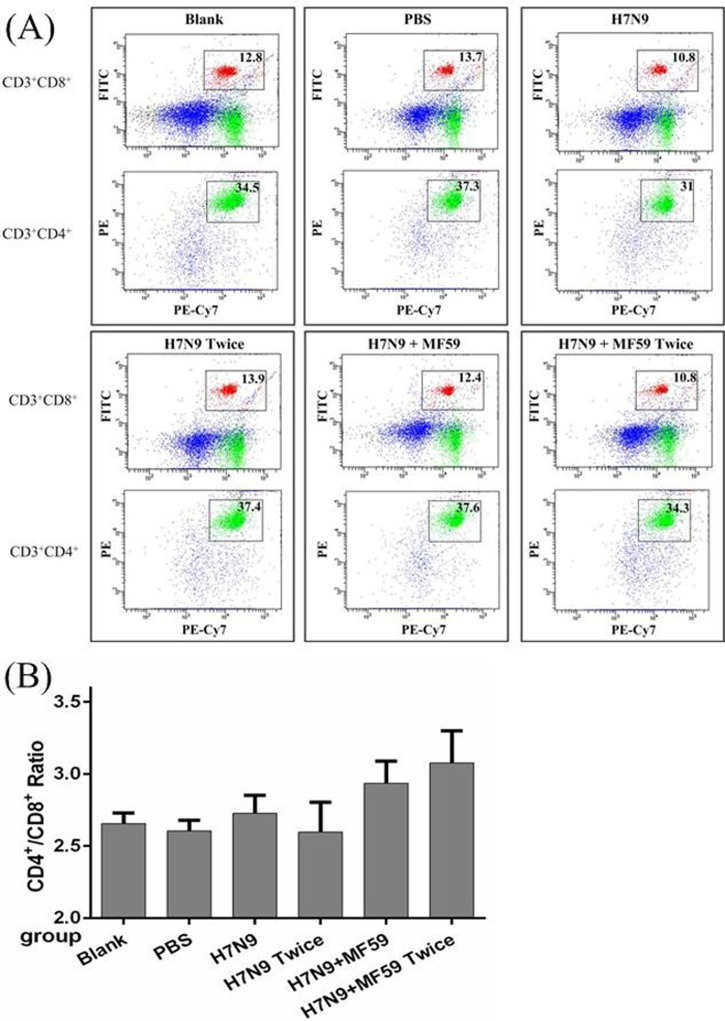
The spenocyte subpopulations of CD3^+^, CD4^+^, and CD8^+^ cells in each group Mice were vaccinated with nothing, PBS, one dose 3 μg HA, one dose 3 μg HA plus 0.05 mL MF59, two doses 3 μg HA and two doses 3ug HA plus 0.05 mL MF59 two weeks after last dose. **A.** Splenocytes were divided into subpopulations based on the surface expression of CD3^+^CD4^+^ (T helper cells), CD3^+^CD8^+^ (cytotoxic T cells). **B.** The CD4^+^/CD8^+^ ratio in each of the six groups.

The CD4^+^/CD8^+^ ratio of splenocytes was quantified (Figure 3A) and found to increase in the groups treated with the MF59 adjuvant (Groups 5 and 6) compared with the other groups (Groups 1 − 4) (*p* < 0.001); however, there were no significant differences in the CD4^+^/CD8^+^ ratios between the groups that received the H7N9 antigen without the adjuvant (Groups 3 and 4) and those that received the blank and PBS controls (*p* = 0.689) (Figure 3B). In conjunction with the cytokine profile, the MF59 adjuvant was associated with a significant accumulation in CD4^+^ splenocytes.

### Protective effects of the candidate vaccine

**Figure 4:**
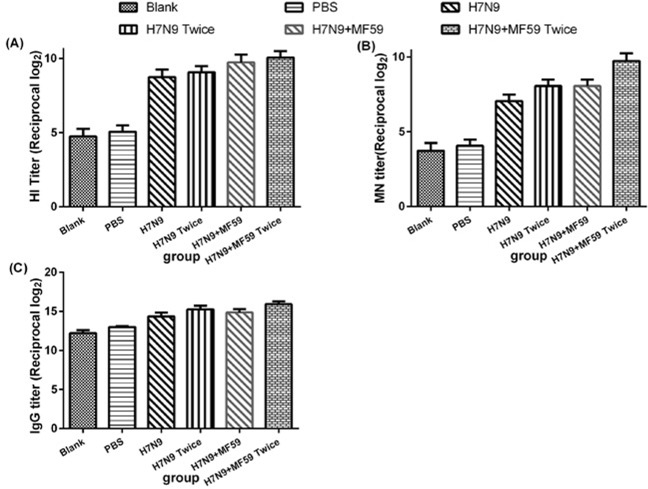
Antibody responses, including A. HI antibodies, B. MN antibodies, and C. IgG titers, for groups of mice one week after viral challenge Each dot represents the geometric mean titer obtained from groups of six mice. The mice were intramuscularly injected with various immune formulations: Groups 1 and 2 were treated with nothing and PBS, respectively. Groups 3 and 4 were immunized with one dose 3 μg HA, one dose 3 μg HA plus 0.05 mL MF59, respectively; Groups 4 and 6 were immunized with two doses 3 μg HA and two doses 3 μg HA plus 0.05 mL MF59, respectively with a two-week interval. Six months after the last immunization, the mice in each group were intranasally inoculated with 50 μL 10^6^ TCID_50_ wild type H7N9 virus A/Zhejiang/DTID-ZJU01/2013 (H7N9).

Even six months after immunizing with the HA antigen (Groups 3 − 6), the antibody titers were significantly increased following viral challenge (Figure 4). Since the antibody response increased progressively with MF59 (Groups 5 and 6), Groups 3 − 6 were compared to evaluate the effects of the MF59 adjuvant (HI titer, *p* = 0.015; MN titer, *p* = 0.023; IgG titer, *p* = 0.045). The comparison between Groups 3 and 5 and Groups 4 and 6 revealed that there was also a substantial impact of a second boost on the antibody response (HI titer, *p* = 0.034; MN titer, *p* = 0.023; IgG titer, *p* = 0.008).

**Figure 5:**
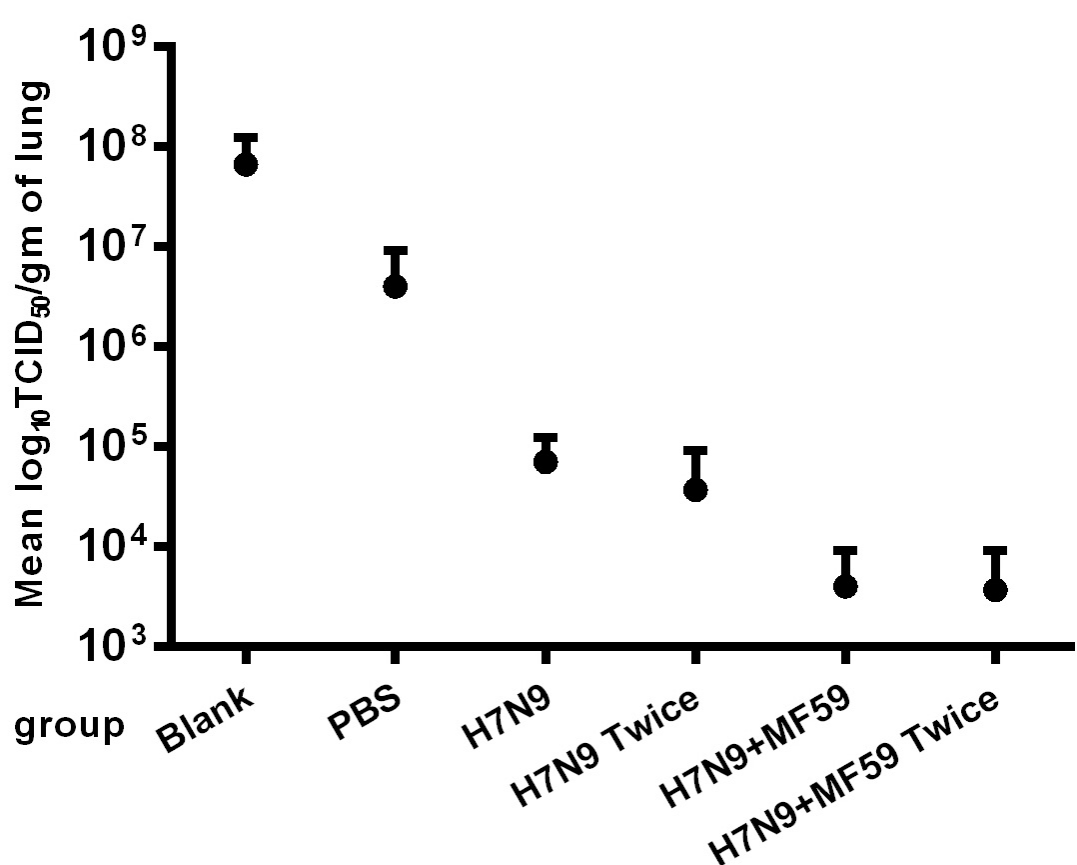
Virus titers in the lungs one week after the H7N9 viral challenge Replications of the H7N9-WT challenged viruses one week after viral challenge in the lungs of immunized BALB/c mice were determined using the TCID_50_ method in MDCK cells. Virus titers in lungs are expressed as the means ± SE of the log_10_ TCID_50_ per g of tissue.

We observed lethargy, rough hair, and a loss of appetite in all six experimental groups after wide type H7N9 challenge but no deaths. Figure 5 presents the viral titers in the lungs one week after H7N9 viral challenge, for which a second boost was found to reduce the viral titers compared with the groups immunized once with no statistical difference (*p* = 0.456). Moreover, the immunogenicity generated in reponse to H7N9 antigen was more effective when paired MF59 adjuvant (*p* = 0.045).

### Histopathological analysis of the H7N9 vaccine with or without adjuvant

**Figure 6:**
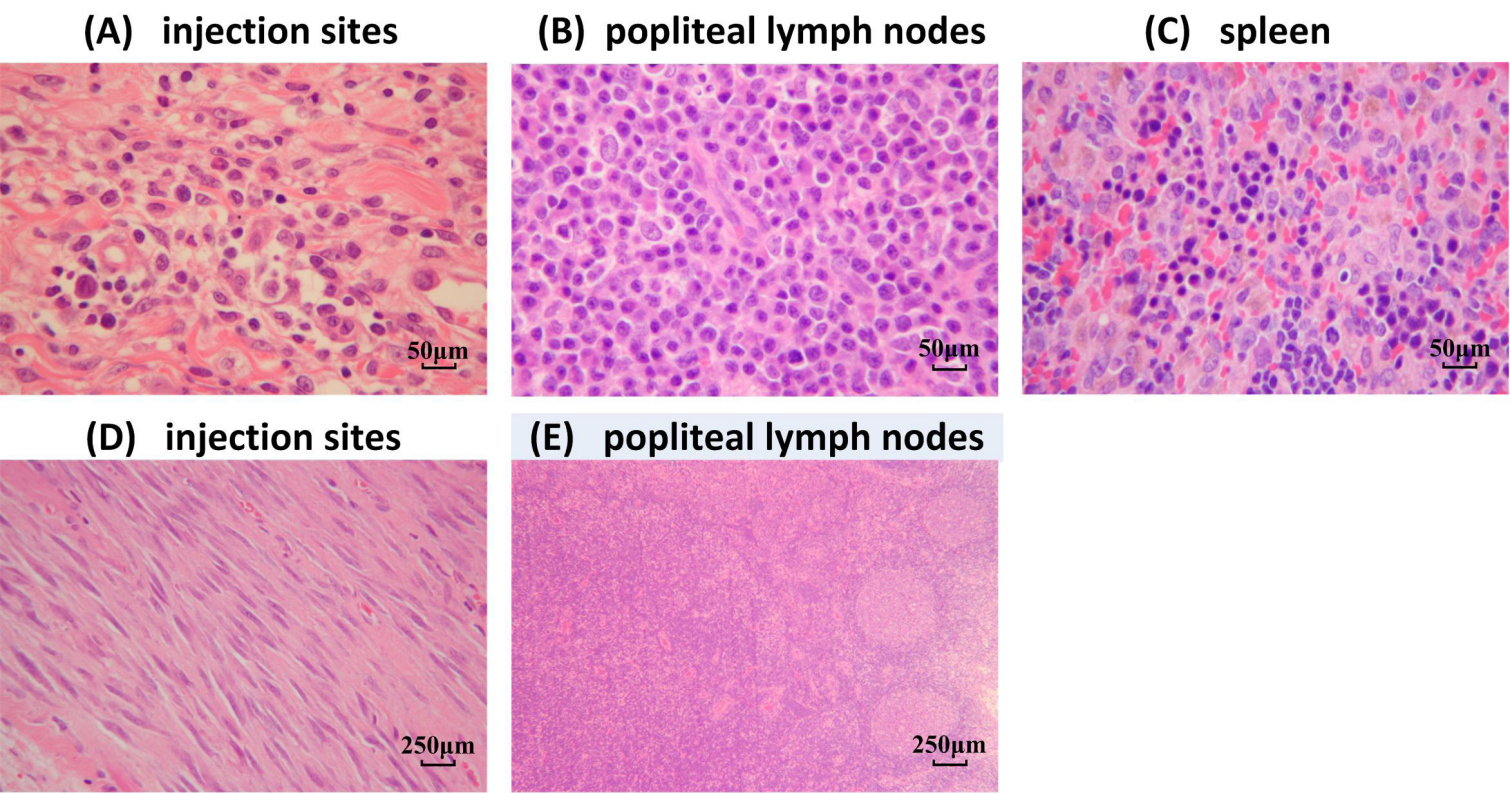
Histological analysis of various tissues H&E staining was performed on sections of various organs from mice immunized with the H7N9 vaccine with the MF59 adjuvant. **A.**-**C.** Two weeks after the last immunization (original magnification × 200). **A.** Intramuscular injection sites, **B.** popliteal lymph nodes, and **C.** spleen. **D.**-**E.** Six months after the last immunization (original magnification × 40). **D.** Intramuscular injection sites and **E.** popliteal lymph nodes.

Two weeks after the final immunization, no inflammatory reactions or other histopathological changes were observed in any of the tissues, including the heart, liver, lung, kidney, stomach, brain, bone, and intestines (data not shown). Immunization with the influenza vaccine with adjuvant (Figure 6A) caused mixed cell infiltration in the interstitium of the intramuscular injection sites. We also observed lymphoid hyperplasia in the follicles and plasmacytosis in the popliteal lymph nodes (Figure 6B). The popliteal lymph node was the local draining lymph node for the injection site, and these changes were expected following vaccine delivery. Extramedullary hematopoiesis in the spleen (Figure 6C) was also observed. This change was considered to be an adaptive response to the inflammatory reaction incited by the injection of the adjuvant. Six months after the last immunization, only mononuclear cell infiltration and fibroplasias at the injection sites (Figure 6D), as well as lymphoid hyperplasia and plasmacytosis in the popliteal lymph nodes (Figure 6E) were noted; albeit most of the changes had resolved.

## DISCUSSION

Studies have established the great significance of vaccination for mitigating an influenza pandemic [15]. For influenza viruses, highly efficient reverse genetics systems that allow the generation of desired reassortants from cloned cDNA directly are now available [16, 17]. This process can remove major molecular features that confer high virulence, which is particularly useful for the highly pathogenic H5 or H7 strains and can be an asset for the rapid preparation of vaccine strains. Our “six plus two” reassorted vaccine seed (the HA and NA gene segments of the A/Zhejiang/DTID-ZJU01/2013 virus on a PR8 genetic background) was produced using eight individual plasmid transfection system. We have demonstrated the low virulence and transmissibility of this strain, indicating that it is suitable for use as an ideal influenza vaccine seed strain. Moreover, this vaccine seed can be parenterally administered in an inactive form to produce the split vaccine. Most H7N9 vaccines are developed based on the A/Shanghai/2/2013 and A/Anhui/1/2013 strains. The A/Zhejiang/1/2013 strain was the first to be used in our split vaccine. The inadequacy of split vaccines is low immunogenicity, because these HAs are poor vaccine antigens [18]. Thus, adjuvants must be employed to augment the immune response to split vaccines. The MF59 emulsion first gained approval for human use in 1997, and was subsequently approved in 23 countries for use as a part of the influenza vaccine for elderly subjects. A previous large-scale analysis supported the good safety profile and the associated substantial immunogenicity [11, 19]. A histopathological examination in this study also demonstrated that the MF59 adjuvant may stimulate a more severe but local and recoverable inflammatory response, which may be related to its strong potentant immune stimulatory effects. We also confirmed that MF59 exerted a more robust response when paired with this split vaccine compared with the most traditional alum adjuvant [20].

In the absence of any established standard of protection to avian influenza, a good correlation was shown between MN titers ≥ 80. The analysis of the sera from patients who recovered from an avian influenza infection also supports the MN titer ≥ 1:80 as potential predictor of clinical benefit [21]. This study confirmed that the vaccination of mice with the two-dose split H7N9 vaccine produced long-lasting antibody titers, with titers (MN titer ≥ 80) persisting for ≥ two months in vaccinated mice. Moreover, the addition of MF59 can raise this value to four months. We found that combining the H7N9 antigen with the MF59 adjuvant and a delivering a second dose increased the immugenicity of the vaccine and ultimately the protective efficacy against H7N9 virus six months post-immunization. In addition, despite almost a disapearance of antibodies from the serum samples of vaccinated mice, humoral response including HI and MN antibodies and IgG titers progressively increased following viral challenge. Furthermore, the histopathology in the lungs also revealed that two doses of the MF59 adjuvanted H7N9 vaccine provides substantial protection for at least 6 months.

Helper T cell (Th) responses can be induced by a particular adjuvant and secrete a spectrum of cytokines [22]. Secretion of IFN-γ and IL-2 (a Th1-type response), has been typically associated with CTL and delayed-type hypersensitivity responses. In contrast, Th2-type responses which are characterized by the secretion of IL-4, IL-5, and IL-10 may invoke B cell activation and antibody production [23]. Additionally, it is well established that different adjuvants used in a vaccine can bias either a Th1 or a Th2 response. Low levels of the cytokines IL-4, IL-5, and IL-10 were detected in all six groups at two weeks, and one, two, four, and six months following immunization, this finding may not be surprising given the relatively short half-life of cytokines in mice, presumably due to its rapid clearance by receptor-mediated uptake. One limitation of this study is that we did not perform a short-term analysis of cytokine changes (in hour increments following immuization) or determine the specific IgG subclasses that were induced by the vaccine. Following viral challenge, there was a substantial increase in Th-2 cytokines, including IL-4, IL-5 and IL-10, detected in the serum of mice immunized with two doses of the MF59 adjuvant H7N9 split vaccine. These findings indicate that the MF59 adjuvant-induced serum cytokine profiles are consistent with a primarily Th2-type response and a second dose can boost this effect. In addition, we found that adding MF59 to the split vaccine caused a reduction in the IFN-γ response and an increase in the IL-2 response following the viral challenge, which is consistent with previous results indicating that MF59 adjuvants are poor inducers of Th1 responses [23]. Th2-type responses reported to invoke a humoral immune response and were consistent with the production of antibodies, while high concentrations of IFN-γ and can be generally immunosuppressive and most Th1 clones are directly cytotoxic for activated B cells and antibody production [24]. Moreover, CD4^+^ cells haves been shown to be beneficial for the early clonal expansion of B cells and for the generation and memory B cells [25, 26], and recent advances have suggested a close association between CD4^+^ T cell and antibody responses [27]. Thus, in conjunction with the observed cytokine profile, we verified that MF59 can promote CD4^+^ activation.

In conclusion, a monovalent H7N9 split vaccine was manufactured based on the A/Zhejiang/1/2013 strain using reverse genetics. All previously published articles regarding H7N9 vaccines have evaluated only the short-term efficacy over duration of a few weeks [28, 29]. In this study, we prolonged the observation to six months and carried out this preclinical experiment in a relatively comprehensive and systematic manner. We observed antibody fluctuation and changes in the levels of serum cytokines in the six months following immunization, and subsequently evaluated the protective efficacy and possible immune mechanisms of the vaccine. In mice immunized with the H7N9 split vaccine, both antibody production and Th2 responses were strongly boosted by the MF59 adjuvant and a second dose. Such responses may be strongly associated with activation of the Th2 response as two doses of the MF59 adjuvant H7N9 split vaccine were found to elicit protective antibody responses which persisted for at least four months, and protected mice from subsequent Zhejiang virus challenge as long as six months after immunization. In addition, MF59 was able to stimulate endogenous CD4^+^ T cell activation.

## MATERIALS AND METHODS

Six to seven-week-old female BALB/c mice were purchased from Joint Ventures SIPPER-BK Experimental Animal Co. (Shanghai, China). All animal studies were performed in accordance with the Guide for the Care and Use of Laboratory Animals of Zhejiang Province and were approved by the local Ethics Committee. Zhejiang Tianyuan Bio-Pharmaceutical Co., Ltd. (which was affiliated with Novartis Vaccine Inc.) provided the MF59^TM^ adjuvant (MF59 is a trade mark of Novartis AG and Affiliate Companies). The Madin-Darby canine kidney cell line (MDCK) was obtained from ATCC (Manassas, VA, USA).

### Viruses and vaccines

The A/Zhejiang/DTID-ZJU01/2013(H7N9) virus was isolated from a patient in Zhejiang province, China in 2013. By transfecting eight individual pHW2000 plasmids into Vero cells [16], we created a A/ZJU01/PR8/2013 vaccine seed strain which harbored HA and NA genes from the aforementioned virus and six internal genes from the PR8 virus, then this reassortant virus was propagated in specific-pathogen-free (SPF) embryonated chicken eggs. The following processes were required before acquiring the split vaccine: clarification, ultrafiltration, zonal centrifugation, purification, cleavage, extinguishing, the second- zonal centrifugation, sterile filtration and identification. Finally, the HA content was quantified using one-way immune diffusion.

### Animal immunization

**Figure 7:**
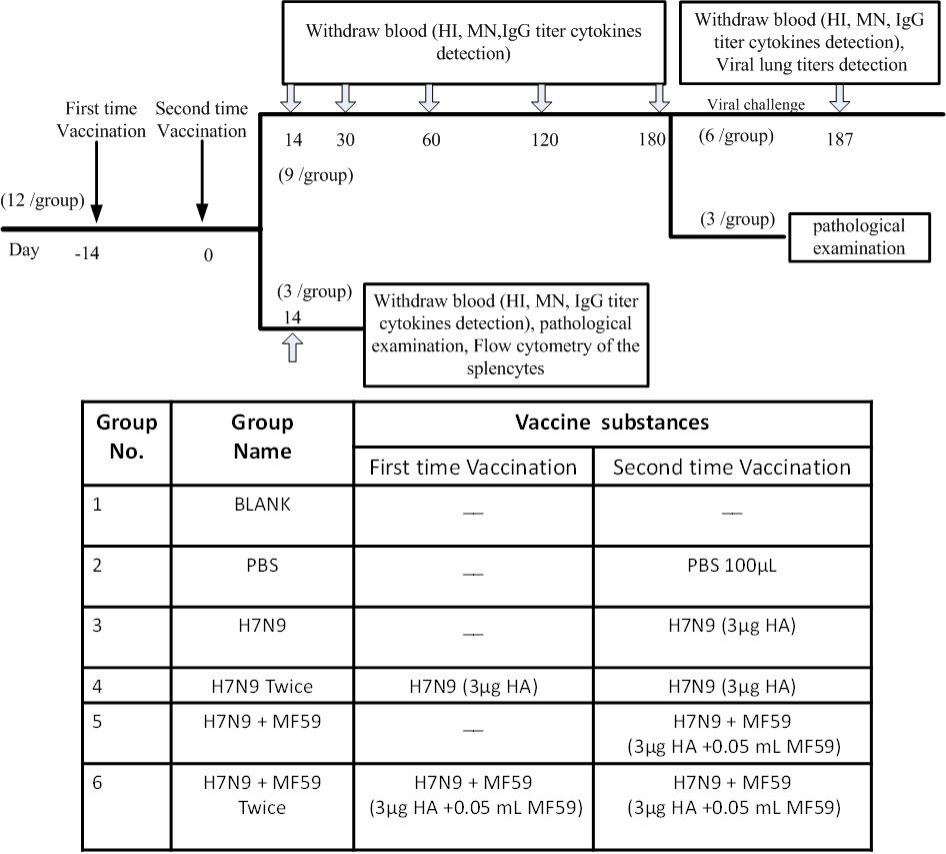
Flow chart (top) and table (bottom) of experimental design Arrows “↓” indicate days of vaccination and Arrows “
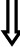
” indicate days of sampling. HI: hemagglutinin inhibition; MN: Micro-neutralization; IgG: Immunoglobulin G.

Groups (*n* = 12) of six to seven-week-old female BALB/c mice were immunized twice with 100 μL of different immune formulations *via* an intramuscular injection in the hind legs (half dose per injection site; i.e., in each hind leg). As shown in Figure 7, Groups 1 and 2 were treated with nothing and PBS, respectively. Groups 3 − 6 were immunized with 3 μg HA, 3μg HA, 3 μg HA plus 0.05 mL MF59, and 3 μg HA plus 0.05 mL MF59, respectively, in 100 μL PBS. Groups 2, 4, and 6 were boosted with PBS, 3 μg HA and 3 μg HA plus 0.05 mL MF59, respectively by a 2-week interval. All animals were bled *via* the tail vein the day before immunization, at two weeks, one month, two months, four months, and six months thereafter, and again one week following viral challenge. A lack of pre-existing immunity against the influenza virus was ascertained in all mice (HI activity < 10).

### Virus inoculation

Six months after the last immunization, mice in each group (*n* = 6) were intranasally inoculated with 50 μL 10^6^ TCID_50_ wild type H7N9 virus A/Zhejiang/DTID-ZJU01/2013(H7N9) diluted in PBS. A TCID_50_ assay was performed using the method of Reed and Muench [30]. Mice were observed for illness and death for seven days after infection.

### HI test

All serum samples were treated with receptor destroying enzyme [9]. Prior to HI testing, a two-fold serial dilution series of serum was mixed 1:1 with four hemagglutinating units of virus (wild type H7N9 virus A/Zhejiang/DTID-ZJU01/2013 (H7N9)) and incubated at 37°C for 1 h. Subsequently, 50 μL 1% chicken erythrocytes were added, mixed, and incubated for 1 h at 4°C; all agglutination patterns were read within 10 min.

### MN assay

MDCK cells were seeded at a density of 2 × 10^4^ cells/well in 96-well plates and cultured to 80%-90% confluency at 37°C. Heat-inactivated serum samples were diluted 1:10 with DMEM, subjected to a two-fold serial dilution, and mixed with 50 μL of 10^6^ TCID_50_ A/Zhejiang/DTID-ZJU01/2013(H7N9) virus for 1 h at 37°C. Cells were incubated in the presence of TPCK-treated trypsin at 37°C for 72 h post-infection. Cell supernatants were then harvested and transferred to V-bottom 96-wells plates. The presence of virus was detected using a hemagglutination assay.

### Immunoglobulin G enzyme-linked immunosorbent assay (IgG-ELISA)

All 96-wells of polyvinyl chloride microtiter plates (Falcon, USA) were coated overnight at 4°C with 100 ng/well HA antigen in a PBS coating solution (KPL, USA). At room temperature, the wells were coated with 1% bovine serum albumin (Sigma-Aldrich, USA) and incubated for 2 h after three washes. Next, the wells were washed and two-fold serial dilutions of serum were inoculated in 100 μL volumes for 1 h. The plates were washed once again, and 100 μL peroxidase-labeled rabbit anti-mouse immunoglobulin G (Zymed, USA) was added to each well and incubated for 2 h. After another wash, 100 μL/well substrate TMB (Sigma) was added and the reaction was subsequently stopped. Absorbance was measured at 450 nm. ELISA titers were expressed as reciprocal dilutions and yielded an OD that was higher than the average OD of the blanks plus three times the SD.

### Immunoassay-luminex measurement

Serum samples were measured using multiplex bead assays (#M6000003J7, Bio-Rad; USA) incorporated into MILLIPLEX MAP panels that were run on a Luminex 200 instrument. All samples were thawed on ice, vortexed, centrifuged at 14, 000 × *g* for 10 min at 4°C, diluted 1:4, followed by vortexing of all bead-antibody complexes. To each well, 50 μL of bead-antibody complexes was added and then washed followed by the addition of either 50 μL of the standard, controls, or samples. Plates were incubated at room temperature for 30 min with constant agitation, then washed three times and incubated for 30 min with 25 μL biotinylated detection antibody. Next, the plate was washed and 50 μL streptavidin-PE was added to the detection antibody for another 10 min. Finally, plates were washed and the beads were suspended in 125 μL assay buffer. For analysis, the 96-well plate was placed in a BioPlex reader and the data collection, analysis, and quality control were performed as previously described by Lovestone [31].

### Flow cytometry of the splenocytes

Spleens were aseptically removed from the mice two weeks after the last immunization and gently dissociated through a stainless-steel sieve into RPMI medium supplemented with 5% fetal calf serum, and collected by pelleting at 1, 200 rpm in a Beckman GPKR centrifuge. Erythrocytes were lysed for 1 min in 155 mM NH4Cl-17mM Tris-HCl (pH 7.2). The cell number was determined in a Neubauer chamber.

Splenocytes were studied by a standard protocol for analysis *via* flow cytometry. Suitable amounts of conjugated monoclonal antibodies against CD3^+^/4^+^/8^+^ were added to 0.1 mL splenocytes and incubated for 30 min at room temperature. Preparations were then washed and fixed using the FACS lysing solution (Becton Dickinson Immunocytometry System, Mountain View, CA, USA). The following combinations of monoclonal antibodies were used: PE-Cy™7-CD3^+^, PE-CD4^+^ and FITC-CD8^+^ (BD PharMingen, San Diego, CA) for recognizing T lymphocytes, T helper, and cytotoxic T lymphocytes, respectively.

### Virus titers of lung tissues

Viral lung titers were determined using 10-fold serial dilutions of tissue extracts, and were tested for infectivity of MDCK cells in 96-well plates after 48 h incubation. Viral titers were estimated based on the method of Reed and Muench.

### Analysis of vital organs by histopathology

All vital organs, including the heart, liver, spleen, lung, kidney, stomach, brain, bone, intestines, popliteal lymph node, and injection site of three mice from each group were harvested at two weeks and six months after the last immunization. After the tissues were fixed and embedded in paraffin wax, 4-μm thick sections were prepared, stained with H&E, and examined microscopically.

### Statistical analysis

Statistical analyses of the data for HI, antibody, and viral titers were performed with SPSS software (SPSS Inc., USA) using a two-way ANOVA test with a Turkey post-hoc assessment. Values represent the means ± SEM for the indicated sample sizes. A threshold of *p* < 0.05 was used to denote statistical significance.

## Abbreviations

HA: hemagglutinin
NA: neuraminidase
DMEM: Dulbecco modified Eagle medium
HI: hemagglutinin inhibition
MN: Micro-neutralization
IgG: Immunoglobulin G
SPF: specific-pathogen-free
PR8: A/PuertoRico/8/34(H1N1)
H7N9: avian influenza A (H7N9)
